# Supplementary material for: Paper-Based Interleukin-6 Test Strip for Early Detection of Wound Infection
Source: Biomedicines. 2022 Jul 3;10(7):1585. doi: 10.3390/biomedicines10071585 (PMC9313325; doi:10.3390/biomedicines10071585)
Supplement: Supplementary file 1 [file biomedicines-10-01585-s001.zip › biomedicines-1746122-supplementary.pdf]

Supplementary Table S1. Patient information, tissue IL-6 concentrations (conventional ELISA and IL-6 test strip), systemic CRP levels and microorganisms and pathological findings

Sample ID	Patient No.	Age (y/0)	Sex	Sample type	CRP (mg/L)	Plate ELISA (IL-6, pg/mL)	Paper-based ELISA (IL-6, pg/mL)	Infection	microorganism or pathology
1	1	67	M	Acute	364.4	12.02		Yes	Escherichia coli, Enterococcus faecalis
2	1	67	M	Acute	25.9	96.95		Yes	Acinetobacter pittii, Escherichia coli
3	1	67	M	Acute	129.8	242.72	200.48	Yes	Klebsiella pneumoniae
4	2	57	M	Acute	230	27.78		Yes	Morganella morganii, Klebsiella pneumoniae, Proteus vulgaris, Vagococcus fluvialis
5	2	57	M	Acute	12.2	20.29		No	-
6	3	33	M	Acute	24.3	16.71		No	-
7	4	73	M	Acute	1.6	34.5		No	-
8	4	73	M	Acute	1.4	7.56		No	-
9	4	73	M	Acute	2.6	2.59		No	-
10	4	73	M	Acute	7.1	8.66		No	-
11	5	62	M	Acute	7.7	308.88		Yes	Streptococcus mitis, Streptococcus oralis, Enterococcus faecalis
12	6	59	M	Acute	27.4	19.1		Yes	Streptococcus agalactiae, Klebsiella pneumoniae, Citrobacter koseri
13	7	51	M	Acute	9.9	178.9		Yes	Escherichia coli, Serratia marcescens, Enterococcus faecalis Yeast
14	8	65	M	Acute	61.1	8.86		No	-
15	9	75	F	Acute	29	47.54		Yes	Citrobacter braakii, Enterococcus faecalis
16	9	75	F	Acute	12.9	13.33		No	-
17	9	75	F	Acute	7.9	26.46		No	-

18	10	57	M	Acute	4.5	107.62	363.65	Yes	Tissue necrosis with infection
19	11	67	F	Acute	4	56.9	110.48	Yes	Proteus mirabilis, Enterococcus faecalis
20	11	67	F	Acute	1.3	50.79		Yes	Proteus mirabilis, Enterococcus faecalis
21	11	67	F	Acute	2.5	49.24		Yes	Proteus mirabilis, Enterococcus faecalis
22	12	73	F	Acute	9.8	690.22	265.42	Yes	tissue necrosis
23	13	67	M	Acute	110.6	1892.65	794.69	Yes	Klebsiella pneumoniae, Enterococcus faecium, Pseudomonas aeruginosa, Yeast
24	14	51	M	Acute	111.3	1558.5	569.49	Yes	Acute and chronic infection
25	15	62	M	Acute	55.4	1996.35	427.15	Yes	Klebsiella pneumoniae, Lactococcus lactis, Lactobacillus fermentum
26	16	40	F	Acute	44.7	53.75		Yes	Pseudomonas aeruginosa, Serratia marcescens, Paenibacillus cookii
27	17	39	M	Acute	32	35.06	30.90	Yes	Enterococcus faecalis, Corynebacterium striatum
28	18	58	F	Acute	53.8	5664.27	2951.92	Yes	Micrococcus luteus
29	19	41	M	Acute	6.9	963.19	262.40	Yes	Pseudomonas stutzeri
30	20	89	F	Acute	102.8	1980.99	2084.64	Yes	Acinetobacter baumannii(XDRAB)
31	21	64	M	Acute	94.8	19.83	1.08	Yes	Proteus mirabilis, Enterococcus faecalis, Pseudomonas aeruginosa
32	22	52	M	Chronic	9	16		Yes	Pseudomonas aeruginosa
33	22	52	M	Chronic	10.8	1.1		No	-
34	23	56	M	Chronic	34.8	28.62		No	-

35	23	56	M	Chronic	2.7	17.81		No	-
36	24	68	F	Chronic	110.8	388.2		Yes	Escherichia coli, Streptococcus dysgalactiae
37	24	68	F	Chronic	44.0	17.43		No	-
38	24	68	F	Chronic	3.5	26.95		No	-
39	25	68	M	Chronic	164	15.02		Yes	Actinomyces species, Campylobacter ureolyticus
40	25	68	M	Chronic	172.3	36.44		Yes	Gangrene with infection
41	25	68	M	Chronic	96	12.26		Yes	Escherichia coli, Klebsiella oxytoca, Enterococcus faecalis
42	25	68	M	Chronic	79.3	3.29		No	-
43	25	68	M	Chronic	93	13.63		No	-
44	25	68	M	Chronic	86.4	6.07		No	-
45	25	68	M	Chronic	41.9	32.6		Yes	Escherichia coli
46	25	68	M	Chronic	4.4	3.68		No	-
47	26	72	M	Chronic	2.8	10.33		No	-
48	27	57	M	Chronic	16.8	12.86		No	-
49	28	61	F	Chronic	112	8.26		Yes	Staphylococcus aureus(ORSA), Pseudomonas aeruginosa
50	28	61	F	Chronic	43.9	25.76		Yes	ORSA
51	29	60	F	Chronic	2.0	63.4		Yes	Tissue necrosis with infection
52	30	37	M	Chronic	61	19		No	-
53	30	37	M	Chronic	17.4	19.6		No	-
54	30	37	M	Chronic	19.5	24.87		No	-
55	31	71	M	Chronic	2.8	86.52		Yes	Acute suppurative infection
56	31	71	M	Chronic	50.3	16.31		No	-
57	32	89	M	Chronic	101	12.5		No	-
58	33	85	F	Chronic	67.4	74.7		Yes	Escherichia coli, Klebsiella pneumoniae
59	34	64	M	Chronic	281	25.91		No	-
60	34	64	M	Chronic	13.9	224.76	252.82	Yes	Suppurative inflammation

61	34	64	M	Chronic	9.4	38.73	18.28	Yes	Pseudomonas aeruginosa
62	35	82	F	Chronic	11.7	36.63		Yes	Tissue necrosis with infection
63	35	82	F	Chronic	13.8	679.48	166.29	Yes	Tissue necrosis with infection
64	36	24	M	Normal	0.5	8.53		No	-
65	37	25	F	Normal	22.5	14.9		No	-
66	37	25	F	Normal	0.8	21.09		No	-
67	38	55	F	Normal	4.2	9.37		No	-
68	39	71	M	Normal	371.5	21		No	-
69	40	61	M	Normal		20.6		No	-
70	41	51	M	Normal	0.8	20.2		No	-
71	42	67	M	Normal		54.8		No	-
72	43	47	F	Normal	190.8	31.9		No	-
73	44	61	M	Normal	0.9	0.9		No	-
74	45	70	F	Normal		3.29		No	-
75	46	50	F	Normal		42.77		No	-
76	47	55	M	Normal		19.5		No	-
77	48	49	M	Normal		18.6		No	-
78	49	55	M	Normal		0.8		No	-
79	50	62	M	Normal		3.49		No	-
80	51	32	F	Normal		32.23		No	-
81	52	58	M	Normal		1.2		No	-
82	53	38	F	Normal		25.5		No	-
